# A Review of the Molecular Mechanisms Underlying the Development and Progression of Cardiac Remodeling

**DOI:** 10.1155/2017/3920195

**Published:** 2017-07-02

**Authors:** Leonardo Schirone, Maurizio Forte, Silvia Palmerio, Derek Yee, Cristina Nocella, Francesco Angelini, Francesca Pagano, Sonia Schiavon, Antonella Bordin, Albino Carrizzo, Carmine Vecchione, Valentina Valenti, Isotta Chimenti, Elena De Falco, Sebastiano Sciarretta, Giacomo Frati

**Affiliations:** ^1^Department of Medical Surgical Sciences and Biotechnologies, “La Sapienza” University of Rome, Latina, Italy; ^2^Department of AngioCardioNeurology, IRCCS Neuromed, Pozzilli, Italy; ^3^Department of Medicine, Washington University School of Medicine, St. Louis, MO, USA; ^4^Department of Medicine and Surgery, University of Salerno, 84081 Baronissi, Italy; ^5^IRCCS, Bambino Gesù, Rome, Italy

## Abstract

Pathological molecular mechanisms involved in myocardial remodeling contribute to alter the existing structure of the heart, leading to cardiac dysfunction. Among the complex signaling network that characterizes myocardial remodeling, the distinct processes are myocyte loss, cardiac hypertrophy, alteration of extracellular matrix homeostasis, fibrosis, defective autophagy, metabolic abnormalities, and mitochondrial dysfunction. Several pathophysiological stimuli, such as pressure and volume overload, trigger the remodeling cascade, a process that initially confers protection to the heart as a compensatory mechanism. Yet chronic inflammation after myocardial infarction also leads to cardiac remodeling that, when prolonged, leads to heart failure progression. Here, we review the molecular pathways involved in cardiac remodeling, with particular emphasis on those associated with myocardial infarction. A better understanding of cell signaling involved in cardiac remodeling may support the development of new therapeutic strategies towards the treatment of heart failure and reduction of cardiac complications. We will also discuss data derived from gene therapy approaches for modulating key mediators of cardiac remodeling.

## 1. Introduction

Understanding the molecular basis of cardiac remodeling is one of the main challenges in cardiovascular medicine. The term cardiac remodeling was used for the first time by Hockam and Bulkley following the observation of regional dilatation and thinning of infarcted myocardium in rats [1]. Subsequently, Pfeffer et al. used remodeling to describe the volume increase of the left ventricular cavity following myocardial infarction (MI) [2]. Today, this term broadly refers to changes in the heart structure brought on by a variety of pathologic insults, not solely due to myocardial infarction.

Notwithstanding the established role of cardiac remodeling as a cause of ventricular dysfunction, the progression of the events involved in this phenomenon is not fully understood. In fact, multiple factors contribute to the development and progression of cardiac remodeling and LV dysfunction. These factors may have several detrimental overlapping effects affecting cardiac structure and function at multiple levels. For example, cardiac fibrosis may affect both relaxation and contractility. Cardiomyocyte death is a crucial event underlying the development of cardiac dysfunction during stress and determining the progression of cardiac abnormalities overtime. In addition, cardiac hypertrophy and fibrosis and a progressive impairment of contractility and relaxation orchestrate together the detrimental evolution of cardiac remodeling. Several molecular pathways converge in cardiac remodeling. For example, it has been demonstrated that after a cardiac injury, inflammation is sustained through the upregulation of cytokine release, leading to fibroblast proliferation and metalloproteinases activation [3]. Furthermore, oxidative stress and alteration in energy metabolism trigger the hypertrophic and profibrotic signaling cascades, resulting in cell death and progressive cardiomyocyte loss. Inflammation and oxidative stress also directly impair cardiac contractility and relaxation. Similarly, alterations of proteins involved in calcium transport are also responsible for cardiac remodeling, contributing to decreasing systolic and increasing diastolic calcium release and reduced contractility [4]. Additionally, neurohormonal activation, such as the renin-angiotensin aldosterone system, enhances the synthesis of proteins involved in inflammation, cell death, and fibroblast proliferation [5].

Here, we will review the molecular mechanisms involved in cardiac remodeling. We will also describe the experimental evidence that suggest acting on key molecules involved in these dysregulated pathways may improve cardiac outcomes.

## 2. Definition of Cardiac Remodeling in Heart Failure

**Figure 1:**
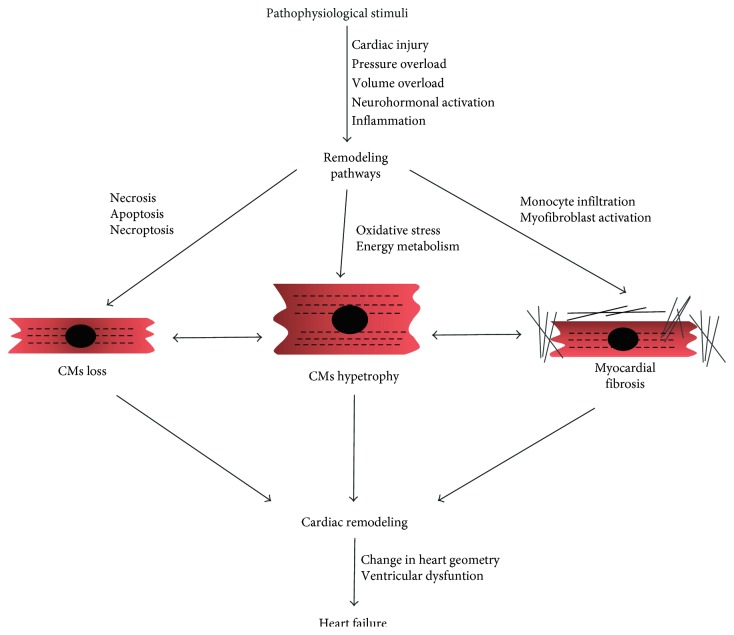
Schematic overview of the main events that contribute to cardiac remodeling. Among the multiple signaling pathways involved, the increase in cell death, inflammation, and oxidative stress pathways, as well as alterations in energy metabolism, converge in cardiomyocyte (CMs) loss, hypertophy, and myocardial fibrosis, leading to cardiac remodeling. The main consequence in such structural modifications is heart failure.

Heart failure (HF) is a chronic heart disease that represents one of the leading causes of mortality worldwide. The term HF usually refers to the inability of the heart to maintain the blood flow necessary to satisfy the metabolic requirements of the body [6]. Cardiac remodeling is strictly associated with the progression of HF [7]. It encompasses all the molecular, cellular, and interstitial events that contribute to the clinically relevant changes in the shape, size, and mass of the heart after cardiac injury [7]. Cardiac remodeling may occur following several pathophysiological stimuli leading to a reduction of contractility and/or an increase in wall stress, such as ischemia/reperfusion (I/R), MI, pressure and volume overload, genetic background, hypertension, and neuroendocrine activation [7–9]. It may be either an adaptive or a maladaptive mechanism [7]. In the first case, structural changes of the heart exert a compensatory effect, maintaining normal cardiac function [10, 11]. On the contrary, after sustained stress, cardiac remodeling leads to a progressive and irreversible dysfunction of the heart [12]. From a cellular point of view, major mechanisms that contribute to cardiac remodeling involve both cardiomyocytes and noncardiomyocytes. In fact, during cardiac remodeling, cardiomyocyte loss has been extensively described to occur through necrosis, necroptosis, apoptosis, or autophagy, whereas fibrosis occurs through fibroblast proliferation and extracellular matrix (ECM) reorganization. Furthermore, mitochondrial dysfunction and metabolic abnormalities also contribute to the development and progression of cardiac remodeling by reducing contractility (Figure 1) [13]. The molecular players and the involved signaling pathways will be discussed in detail below.

Dysregulation of physiological mechanisms, such as excitation-contraction coupling (ECC), a process that tightly regulates calcium influx and uptake, is a common feature of several pathophysiological cellular alterations in cardiac remodeling. In fact, in a failing cardiomyocyte, there is impaired calcium uptake, mediated by proteins such as sarco/endoplasmic reticulum Ca^2+^-ATPase (SERCA)-2a, and uncontrolled calcium efflux through ryanodine receptors (RyRs) [4]. Calcium dysregulation, beyond early macroscopic effects of systolic dysfunction and arrhythmias, can interfere with processes such as hypertrophic growth, energy metabolism, mitochondrial function, and cell survival [13]. These alterations are manifested by changes in heart geometry from an elliptical to a spherical shape, which in turn contributes to impair the contractile function of the heart. Furthermore, cardiac remodeling is characterized by increased left ventricular (LV) mass with a reduction in LV ejection fraction [7, 14].

## 3. Cardiac Hypertrophy

**Figure 2:**
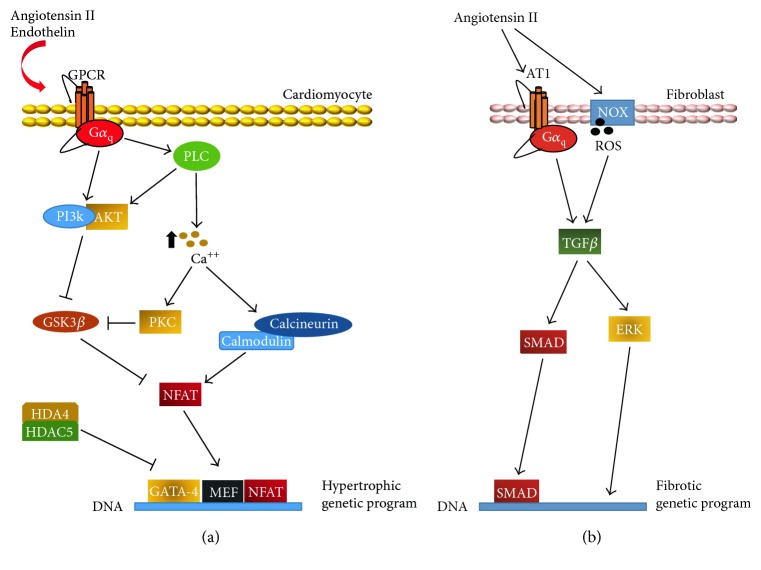
Cardiac hypertrophy (a) and cardiac fibrosis (b) signaling pathways. Several molecules participate in the modulation of genes involved in cardiac hypertrophy. The transcription factor NFAT, responsible for cardiac hypertrophy, is positively regulated through calmodulin/calcineurin. In contrast, GSK3*β* inhibits cytoplasm-nucleus translocation of NFAT. HDAC4/HDAC5 also represses transcriptional activity of hypertrophic signals. Angiotensin II is the main mediator of cardiac fibrosis; AT1 receptor and ROS lead to TGF*β* activation. This latter, through a SMAD-dependent or -independent pathway, activates the fibrotic genetic program, which consists in fibroblast proliferation, leukocyte infiltration, matrix degradation, collagen deposition, and myofibroblastic transdifferentiation.

As a compensatory adaptive response to mechanical and physiological stress impairing cardiac output, cardiomyocytes may undergo hypertrophy (Figure 2). The hemodynamic overload on cardiac walls activates complex biological responses that culminate in tissue remodeling. Tissue remodeling initially starts as compensatory LV hypertrophy, but eventually evolves into maladaptive remodeling, triggering the transition to heart failure [15]. In fact, it was recently shown in three different in vivo animal models of pressure overload that silencing Stromal Interaction Molecule (STIM)-1, one of the molecular initiators of the hypertrophic compensatory response, prevents as well as reverses cardiac hypertrophy. However, the consequence for the animal is a rapid transition to heart failure. Mechanistically, hypertrophic stress leads to increased Ca2^+^ influxes mediated by the association of calcium release-activated calcium channel protein (ORAI)-1/3 with STIM-1, which then activates the mammalian target of rapamycin complex (mTORC)-2. In this study, silencing of STIM-1 was shown to prevent mTORC-2 phosphorylation of Akt kinase, thereby preventing suppression of GSK-3*β* activity, ultimately resulting in inhibition of hypertrophic responses [16].

On the other hand, chronic hypertrophy has been associated with interstitial fibrosis and cellular apoptosis. A precise balance of muscle growth, inflammation, and angiogenesis is necessary to ensure adaptive hypertrophic remodeling; alterations to this equilibrium result in deterioration of cardiac structure and function. During maladaptive cardiac remodeling, sarcomere addition is performed in series—that is, end-to-end—which gradually decreases cardiomyocyte force production leading to contractile dysfunction, ventricular dilation, and arrhythmias [15]. Moreover, several cytokines and growth factors have been found to play a key role in the remodeling of the ventricular chambers in response to hemodynamic overload. For example, placental growth factor (PlGF) has recently been shown to regulate tissue inhibitor of metalloproteinase TIMP-3/TNF-*α*–converting enzyme (TACE) axis during cardiac remodeling in response to overload. In particular, PlGF may act as a transcriptional regulator of TIMP-3 to modulate inflammation, which upregulates cardiac remodeling [17].

Studies on calcium regulation have contributed to a deeper understanding of the physiologic alterations underlying cardiac remodeling. Generally, after contraction is induced by Ca^2+^, the cell actively pumps calcium ions outside the cytoplasm to generate a new gradient. This is achieved through the activity of the sarcoplasmic reticulum (SR) Ca^2+^ pump, sarcolemmal Ca^2+^ ATPase, and the Na^+^/Ca^2+^ exchanger, which utilizes the gradient created by the Na^+^/K^+^ pump. Defects in these mechanisms may result initially in diastolic dysfunction. However, arrhythmias and systolic dysfunction also occur due to impaired excitability and contraction, ultimately leading to cardiac hypertrophy [18, 19].

Mechanical wall stress activates mechanosensitive ion channels, mainly responsible for the heart's responses to acute changes, and integrins, surface proteins intimately connected to the ECM. Signals sensed in this way are then transduced through the Akt pathway. Short-term activation of the Akt pathway was shown to induce LV hypertrophy without affecting cardiac function whereas long-term activation leads to heart failure [20]. In this regard, conditional mutant mice with activated Akt showed reversible cardiac hypertrophy when the inducing treatment was interrupted within 2 weeks of stimulation, whereas further stimulation caused irreversible remodeling, fibrosis, and eventually HF [21]. Akt exerts size control through the inhibitory phosphorylation of GSK3*β*, a negative controller of cellular size. In a genetic mouse model of familial hypertrophic cardiomyopathy (FHC) based on mutated sarcomeric proteins, GSK3*β* was highly phosphorylated [22]. In contrast, mice with GSK3*β* overexpression had reduced heart size in response to stress [23]. Mechanical stretch promotes the release of several factors, including angiotensin (AT)-II and endothelin-1, which converge towards the activation of G-protein coupled receptor (GPCR) signaling through G*α*_q_ subunits. Genetic manipulation of G*α*_q_ was associated with modulation of the hypertrophic phenotype. For example, activation of phospholipase C (PLC), a downstream effector of G*α*_q_, leads to hypertrophy through the PI3K/Akt pathway [24]. Indeed, GSK3*β* exerts its function by inhibiting nuclear translocation of the nuclear factor of activated T-cells (NFAT), an upregulator of hypertrophy. Calmodulin, a kinase that reacts to Ca^2+^ dysregulation by activating the serine/threonine phosphatase calcineurin (a functional antagonist of GSK3*β*), also converges towards NFAT translocation [25].

Within the complex downstream network activated by hypertrophic stimuli, epigenetics plays a central role as well. In fact, Class II histone deacetylases, HDAC4 and HDAC5, normally interfere with DNA binding of prohypertrophic transcription factors, such as NFAT, myocyte enhancement factor (MEF), and GATA-4. Oxidation or phosphorylation on specific residues, performed by kinases such as Ca^2+^/calmodulin-dependent protein kinase (CaMK)-II, GPCR kinase (GRK)-5, PKC, and PKD, causes a cytoplasmic translocation of Class II HDACs, derepressing prohypertrophic transcriptional activity [26, 27].

Interesting insights have been recently reported on paracrine prohypertrophic signaling provided by endothelial cells during pressure overload-induced cardiac remodeling. For example, Appari et al. [28] showed that complement C1q tumor necrosis factor-related protein (CTRP)-9 deletion and overexpression suppresses or upregulates cardiac hypertrophy, respectively, following transversal aortic constriction (TAC). Mechanistically, this is mediated by phosphorylation of the prohypertrophic transcription factor GATA-4 through extracellular-regulated kinase (ERK)-5. The hypertrophic genetic program includes upregulation of signaling molecules such as brain natriuretic peptide (BNP) and atrial natriuretic peptide (ANP), which decrease blood pressure through diuresis, and of structural proteins such as *β*-myosin heavy chain (*β*-myHC) [15]. Interestingly, despite the existence of a tightly controlled mechanism for regulation of myosin heavy chain *α*/*β* isoform ratio, NFAT activation unbalances the physiological 90% proportion of *β*-myHC. The supposed biological rationale is that this isoform requires less ATP, but also has less contractile ability. In fact, it was shown that expression levels of *β*-myHC are inversely correlated to overall contraction capacity, myocyte shortening, and force generation [29].

Natriuretic peptides, together with nitric oxide (NO), activate cGMP-dependent protein kinase (PKG), exerting an antihypertrophic effect. Preclinical and clinical studies with sildenafil, a cGMP-phosphodiesterase inhibitor that stimulates NO production, have shown beneficial effects in congestive heart failure (CHF) [30, 31]. Interestingly, oxidative stress levels are increased during cardiac injury as well as hemodynamic overload. In fact, it has been recently reported that deletion of the superoxide-producing enzyme NADPH oxidase (NOX)-4 attenuates cardiac hypertrophy after 2 weeks of pressure overload [32].

Lastly, interesting evidence is emerging from a protein that has been extensively studied in oncology, the peptidyl-prolyl cis-trans isomerase NIMA-interacting (PIN)-1. This protein acts as a molecular orchestrator in many different physiological and pathological cellular processes, including hypertrophy. In fact, PIN-1 is upregulated in a model of pressure overload. Moreover, PIN-1 depletion interferes with Akt and mitogen-activated protein kinase (Mek) prohypertrophic signaling. Intriguingly, the same protective effect was obtained by PIN-1 overexpression, suggesting that this protein acts within a tight operative range [33].

## 4. Myocardial Fibrosis

Hypertrophy is often flanked by interstitial and perivascular fibrosis, a phenomenon resulting from the combined effect of inflammation and apoptosis. In fact, pathological remodeling is often the consequence of insufficient capillary density that progressively leads to cell death in the infarcted myocardium after the acute event, as well as in the hemodynamically overloaded heart. In fact, it has been shown that expression of vascular endothelial growth factor (VEGF), which is regulated by transcription factors such as hypoxia-inducible factor (HIF)-1*α* and GATA-4, is impaired in the failing heart [21]. However, the underlying molecular mechanism is controversial, since the inflammation-mediated proapoptotic activity of p53 inhibits HIF-1*α* [34], while ROS produced by NOX-4 (an enzyme under control of inflammatory and neurohumoral signals) seems to be positive drivers of HIF-1*α* activation [35].

Macroscopically, the term myocardial fibrosis refers to the deposition of types I and III collagen, and ECM cross-linking, that together cause altered mechanosensing, stiffening of the chamber walls, and impaired heart elasticity and diastolic function [36]. Additionally, it has been reported that fibrosis impairs contractility and disturbs the chemoelectrical conductance of the heart, leading to arrhythmias, local microfibrillations, and inefficient contraction [37]. The development of fibrosis requires (i) increased synthesis of matrix metalloproteinases (MMPs) due to downregulation of MMP inhibitors [38]; (ii) stimulation of profibrotic mediators, such as TGF-*β*, *α*-smooth muscle actin (*α*-SMA), platelet-derived growth factor (PDGF), and cytokines [39]; (iii) differentiation of fibroblasts into myofibroblasts, which express features of smooth muscle differentiation [40]; and (iv) recruitment of cells of an endothelial origin for endothelial-to-mesenchymal transition (EndMT), generating cells that still express endothelial markers while gaining fibroblast-like characteristics [41]. Indeed, fibroblasts play a critical role in fibrosis. Many inflammatory mediators trigger cellular differentiation towards the myofibroblastic phenotype, characterized by expression of *α*-SMA, proliferation, migration, release of proinflammatory signals, and increased production of ECM remodeling proteins. Nevertheless, many questions remain unanswered about the cellular source of these active cells, as extensively reviewed by Travers et al. [42]. Briefly, given that most myofibroblasts derive from resident inactive fibroblasts, which are extremely prone to activation in response to injury in order to preserve heart function, many mesenchymal cells are thought to transdifferentiate towards the myofibroblast phenotype. Strong evidence has also been provided for perivascular cells differentiating and contributing to fibrosis. When human pericytes were injected into the peri-infarct zone of mice, there was improved cardiac remodeling through the activation of a reparative angiogenic program [43, 44]. Although some studies suggest a role for the transdifferentiation of endotheliocytes, epicardial cells, and circulating bone-marrow-derived stem cells, this is still under debate [37, 45–47]. Further efforts may be required for the identification of novel phenotypic markers that can help clarify the contributions of these cell populations to the myofibroblastic pool found in the remodeling heart.

In both adaptive and maladaptive (chronic) fibrosis, there is extensive monocyte infiltration, enriching the local macrophage population. Their primary role within the myocardium is still an object of debate, but the most accepted theory considers these cells molecular orchestrators of the myocardial inflammatory response, achieved through extensive cytokine interplay between macrophages and lymphocytes [3]. Moreover, endothelial cells can strongly contribute to the profibrotic inflammatory environment by activating a proinflammatory secretory phenotype [48], in addition to directly transdifferentiating into myofibroblasts, as previously mentioned. A significant contribution to local inflammation is also provided by several subpopulations of lymphocytes [49, 50] and mast cells [51, 52], which exert a prominent role in the activation of fibroblasts within the myocardium. Furthermore, cardiomyocytes themselves have both an active and passive role in cardiac inflammation; while cardiomyocytes can activate a profibrotic and proinflammatory secretory phenotype, they are also sensitive to the stimuli they are contributing to, resulting in a complex autocrine network between molecular pathways that can ultimately lead to cell death [53].

From a molecular point of view, many signaling pathways, involving both paracrine and endocrine secretion, are involved in the development of fibrosis in pathological cardiac remodeling (Figure 2). In fact, the renin-angiotensin-aldosterone system (RAAS) is responsible for many pathophysiological modifications that occur in cardiac remodeling [5]. Increased angiotensin-converting enzyme (ACE) levels lead to elevated circulating AT-II, which is a well-known profibrotic mediator [54]. Note that activation of AT-1 receptor stimulates expression of transforming growth factor (TGF)-*β* through both SMAD-dependent and SMAD-independent pathways [55, 56]. Given that TGF-*β* is a pleiotropic mediator, its contribution to fibrosis mainly consists of stimulating transdifferentiation towards a myofibroblastic phenotype and increasing expression of many different protease inhibitors [57]. Recently, in a model of myocardial fibrosis, both AT-II and TGF-*β* were reported to mediate fibrosis by increasing the levels of serpinE2/protease nexin-1, which is responsible for collagen deposition [58]. Moreover, Zhang et al. showed the involvement of focal adhesion kinase (FAK) in the processes of collagen deposition and cardiac fibrosis after myocardial infarction [59]. However, TGF-*β* is not the only molecular mediator of RAAS-induced effects. In fact, proliferation, hypertrophy, and fibrosis have also been linked to the mitogen-activated protein kinase (MAPK) pathway (particularly, ERK1/2, c-Jun N-terminal kinase [JNK], and p38). MAPK in turn interacts and associates with the AT-II/AT-1 complex, epidermal growth factor receptor (EGFR), platelet-derived growth factor receptor (PDGFR), and insulin receptor [60]. In summary, while ERK itself is responsible for a mild response, strong stimuli and ROS-mediated triggering of apoptosis signal-regulating kinase (ASK) can activate JNK and p38 [60]. JNK and p38, together with an activated aldosterone receptor, induce fibroblast matrix deposition, modulate MMPs, and increase TIMP expression to stabilize remodeled ECM [61]. This concerted response relies primarily on the activity of major transcription factors, such as nuclear factor (NF)-*κ*B and activator protein (AP)-1. Consequently, stimulated cells enact a proinflammatory response characterized by paracrine secretion of tumor necrosis factor (TNF)-*α*, which increases proliferation and collagen deposition, as well as IL-1*β*, which promotes degradation and remodeling [3, 5].

These cytokines, along with AT-1, which triggers the activity of NOX through I*κ*B inhibition, are also involved in inflammatory ROS production, further exacerbating phlogosis [60, 62].

During MI, damage-associated molecular pattern (DAMPs) proteins are also released from the myocardium, triggering inflammatory fibrotic cardiac remodeling. DAMPs bound to pattern recognition receptors (PRRs), or with toll-like receptors (TLRs), are crucial for the activation of proinflammatory signaling pathways. Among these, most converge on MAPK phosphorylation, NF-*κ*B and interferon regulatory factor (IRF) nuclear translocation, and “NACHT, LRR and PYD domains-containing protein” (NLRP)-3 inflammasome activation. Consequently, cells increase the production of proinflammatory cytokines, chemokines, and cell adhesion molecules [37]. A specific type of PRR is RAGE, a receptor that binds to advanced glycated end products (AGEs), which are known to activate a proinflammatory expression phenotype [63]. Its concentration was described to correlate with cardiac fibrosis in vivo [64]. Moreover, RAGE deletion decreased inflammation, reduced fibrosis, and ameliorated cardiac fractional shortening in an I/R murine model [65]. Interestingly, in the same study, Volz and colleagues demonstrated that leukocytes infiltrating the myocardium, rather than resident cells, were responsible for AGE-associated adverse inflammatory cardiac response. Despite little being known about the molecular mechanism linking AGEs to fibrosis, there is evidence supporting that TGF-*β* may play a major role [66]. Furthermore, in addition to stimulation of fibroblast proliferation and deposition of types I and III collagen, AGEs contribute to enhancement of ECM accumulation, compromising the heart's diastolic function [67, 68].

All of the aforementioned alterations evoke a cell response through so-called “mechanotransduction.” The term refers to the capacity of each cell to sense its own architecture, and modify expression profiles in response to its alteration [69]. The mammalian sterile 20-like kinase (Mst)-1 pathway functions to integrate physical and biochemical stresses and is crucial in many cardiovascular diseases [70]. In fact, it is sensitive not only to alterations of cell morphology and ECM characteristics, but also to many inflammatory signals [71]. Many of these signals converge on yes-associated protein (YAP), a downstream effector of Mst-1; these signals include oxidative stress and metabolic derangements through AMP-activated protein kinase (AMPK) activity [72, 73]; angiotensin II through GPCR-activated PKA [74]; and cytotoxic stress through mTORC-2 [75]. It has previously been shown that activation of Mst-1 reduces autophagy and cell proliferation and may eventually trigger apoptosis. It is thus not surprising that in an environment such as the fibrotic heart, Mst-1 has been recognized as a major contributor to cardiomyocyte mortality [76–80]. Lastly, microRNAs (miRNAs or miRs), which are small noncoding single-stranded RNAs that serve as key posttranscriptional regulators, have also been implicated in the pathogenesis of several cardiovascular diseases, including myocardial fibrosis [81, 82]. Among the most studied miRNAs directly involved in cardiac fibrosis, miR-133a, miR-29, and the miR-21 families seem to play a pivotal role in the genesis and progression of cardiac remodeling toward cardiac fibrosis. Recent data demonstrate a relationship between miR-133a and collagen 1A1 (Col1A1), suggesting that myocardial fibrosis occurring in Ang-II-dependent hypertension is regulated by the downregulation of miR-133a and miR-29b through the modulation of Col1A1 expression [83]. Notably, myocardial infarction has been associated with downregulation of miR-29 expression in cardiac fibroblasts, via the action of TGF-*β* [83]. The miR-29 family, and in particular miR-29s, directly targets the mRNA of different types of collagens and ECM proteins and has a strong antifibrotic effect in the heart. MiR-21 also plays a clear role in cardiac fibrosis; it promotes fibroblast survival, growth factor secretion, and synthesis of collagens through the regulation of the ERK-MAPK signaling pathway, via the inhibition of sprouty homologue 1. In myocardial infarction, miR-21 activates the TGF-*β*/SMAD pathway via suppression of TGF-*β* receptor III in the ischemic area, enhancing collagen production, upregulating *α*-SMA expression, and facilitating fibroblast differentiation into pathological myofibroblasts [84, 85]. These findings are particularly important in the setting of a possible clinical translation. In fact, it is well established that myocardial miRNA expression can be hampered by the use of antisense RNAs. The development of anti-miRNA therapeutics aimed at reducing or reversing fibrosis is of paramount interest: in this way, miR-29 and miR-21, which are both dysregulated in myocardial remodeling, seem to represent main pathogenetic targets for such an approach.

## 5. Inflammation, Metabolism, and Cardiac Remodeling

**Figure 3:**
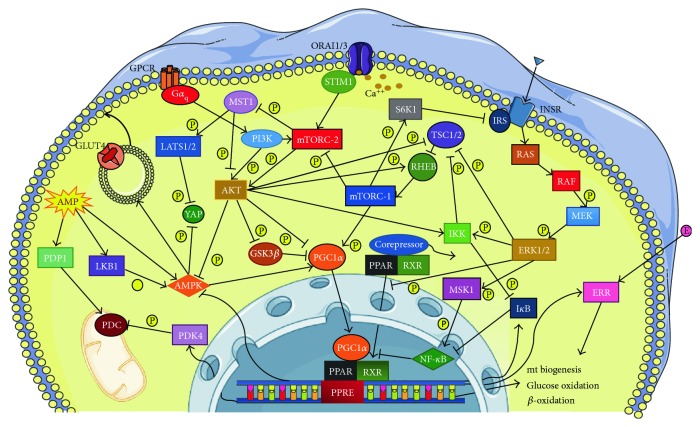
Schematic overview of the relationship between PPAR-response elements (PPREs) and peroxisome proliferator-activated receptor gamma coactivator 1-alpha (PGC1*α*) in cardiac remodelling. AKT: protein kinase B; AMPK: adenosine monophosphate-activated protein kinase; ERK1/2: extracellular signal-regulated kinase 1/2; ERR: estrogen-related receptor; GPCR: G-protein coupled receptor; GSK3*β*: glycogen synthase kinase 3 beta; IKK: I*κ*B kinase; I*κ*B: inhibitor of NF-*κ*B; INSR: insulin receptor; IRS: insulin receptor substrate; LATS 1/2: serine/threonine-protein kinase 1/22; LKB1: liver kinase B1; MEK: mitogen-activated protein kinase kinase; MSK1: mitogen and stress-related kinase 1; MST1: mammalian sterile 20-like kinase; mTORC: mammalian target of rapamycin complex 1 and mTORC-2; ORAI1/3: calcium release-activated calcium channel protein 1/3; PDC: pyruvate dehydrogenase complex; PDK4: pyruvate dehydrogenase kinase; PDP1: pyruvate dehydrogenase phosphatase1; PI3K: phosphoinositide 3 kinase; PI3K: phosphoinositide 3-kinase; RAF: serine/threonine-specific protein kinases; RAS: small GTPase RAS; RHEB: RAS homolog enriched in brain; RXR: 9-cis-retinoic acid receptor; S6 K1: S6 kinase 1; STIM-1: stromal interaction molecule-1; TSC-1/2: tuberous sclerosis- 1/2; YAP: yes-associated protein. See text for details. The figure was made in part using tools provided by Servier Medical Arts.

Chronic inflammation in the remodeling heart reduces ATP and phosphocreatine concentrations, impairing mitochondrial carbohydrate metabolism and fatty acid oxidation [86]. Consequently, the inefficient and acidogenic process of glycolysis meets energy demands anaerobically. At the same time, pharmacological inhibition of fatty acid oxidation ameliorates cardiac function in CHF patients [87]. All these derangements further impair cardiac contractility. In addition, inflammatory cytokines directly reduce contractility by interfering with SERCA2a [88]. While the molecular alterations underlying the development of the so-called “fetal metabolic phenotype” are still the object of debate and intense study, there are several pathways that seem essential in this complex pathophysiological context, such as the axis of peroxisome proliferator-activated receptors (PPARs) and their coactivator PGC-1 (Figure 3). In fact, cardiac metabolism is mostly regulated by the PPAR transcription factor family, whose members recognize specific DNA regulatory sequences, called PPAR-response elements (PPREs). The PPAR family includes three isoforms, PPAR-*α*, PPAR-*β*/*δ*, and PPAR-*γ*, whose relative levels vary in a tissue-specific fashion. In the heart, PPAR-*α* and PPAR-*β*/*δ* are the main isoforms, and previous studies have confirmed their critical role in cardiac metabolism and pathology [89]. PPARs form heterodimers with the 9-cis-retinoic acid receptor (RXR), which has high affinity for many transcriptional corepressors. The binding of the complex with long-chain fatty acids or eicosanoid-derived products induces a conformational change that permits the replacement of the corepressor with a coactivator. In the heart, the best characterized coactivator is PGC-1*α*, which regulates the expression of many genes involved in mitochondrial biogenesis, *β*-oxidation, glucose oxidative metabolism, and the electron transport chain [90]. PGC-1*α* expression is markedly altered in pathologic states; in fact, its level is elevated in conditions of high energy demand [91, 92], but is decreased in heart failure [93], ischemia [94], and hypertrophy [95, 96].

PGC-1*α*, when combined with the PPAR complex, upregulates the transcription of pyruvate dehydrogenase kinase (PDK)-4, a crucial kinase that inactivates the pyruvate-dehydrogenase complex residing on the inner mitochondrial membrane, resulting in decreased glucose oxidation and increased fatty acid utilization [97]. Another major PGC-1*α* target is the estrogen-related receptor (ERR) family, among which ERR*α* drives the expression of genes encoding oxidative phosphorylation and fatty acid oxidation, as well as the PPAR*α* gene itself [98]. Mice overexpressing PPAR-*β*/*δ* display a normal heart, in contrast to those overexpressing PPAR-*α*, which is associated with inflammation [99]. An opposite effect was demonstrated with PPAR cardiomyocyte-specific deficient mice, which manifested a pathologic phenotype only with depletion of isoform *β*/*δ*, but not *α*. Mice lacking the former showed decreased mitochondrial biogenesis, myocardial hypertrophy, and depressed cardiac performance [100]. PGC-1*α* is likewise critically important for controlling processes such as cell metabolism and the inflammatory response. Mice with either overexpression [101] or deletion [102] of this gene develop cardiac abnormalities. Moreover, PPARs have been proposed to physically cross-inhibit inflammatory transcription factors, such as NF-*κ*B, AP-1, signal transducers and activators of transcription (STATs), and NFAT, in a process termed “transrepression” [103]. Furthermore, PPAR-*α* is able to transcriptionally regulate I*κ*B*α*, thus controlling NF-*κ*B activity [104]. Conversely, PPAR-*α* is negatively regulated by MEK-1, an upstream member of the ERK1/2 pathway, which stimulates the nuclear export of PPAR*α* by direct binding [105]. Interference with NF-*κ*B transcriptional activity has also been shown for PPAR-*β*/*δ* [106] and PPAR-*γ* [107, 108].

PGC-1*α* represents a cornerstone of the molecular control in the context of inflammatory cardiac diseases. In fact, NF-*κ*B largely mediates the mechanism by which TNF-*α* downregulates PGC-1*α*. It accomplishes this by acting in concert with the previously described shifts toward different energetic substrates during the progression of cardiac inflammatory pathologies [109]. The physical interaction between NF-*κ*B and PGC-1*α* impairs the latter's capacity to induce its own expression, thereby leading to a reduction of PDK4 expression levels, with a consequent increase in glucose oxidation, as observed during inflammation [110]. It is worth mentioning that the transcriptional capacity of PGC-1*α* may also be compromised through phosphorylation by Akt, which is activated by NF-*κ*B [111]. Finally, PGC-1*α*^−/−^ mice showed lower cardiac power and increased glucose consumption [102], while specific cardiac TNF-*α* overexpressing mice displayed cardiomyopathy and decreased levels of PGC-1*α* and PDK-4 [112].

The PI3K/Akt pathway has been extensively studied in this context as it relates to PPARs. In fact, PI3K mediates many cellular responses in both physiological and pathophysiological states through its effector Akt, which is a core kinase whose down-stream targets include GSK-3*β*, AMPK and mTOR. Akt phosphorylation-mediated inhibition of GSK-3*β* increases cardiac glycogen synthesis [113]. Akt activation has been shown to decrease AMPK activity, which is induced by ATP depletion through phosphorylation by upstream kinases like LKB1. Once activated, it switches off energy-consuming processes and boosts energy-producing pathways. Furthermore, AMPK promotes glucose transporter type (GLUT)-4 expression and translocation to the cell membrane and stimulates glycolytic enzymes. Moreover, AMPK has been reported to be protective against ROS [113]. Additionally, Akt activation stimulates the activity of mTOR kinase, which is responsible for substrate switching and suppression of the inflammatory response. Note that mTOR itself activates Akt and downregulates insulin signaling, inhibiting IRS-1 [114].

## 6. Mitochondrial Dysfunction

All the mechanisms involved in cardiac remodeling may be potentially associated with mitochondrial dysfunction. Recent evidence suggests that mitochondrial dysfunction contributes to the development of several pathologies, including neurodegenerative and cardiovascular diseases [115, 116]. In the heart, the cardiomyocyte mitochondrial compartment is particularly robust to meet energy requests for sarcomere contraction [86]. ATP synthesis occurs through oxidative phosphorylation, a process that relies on electron transfer across multimeric complexes on the inner mitochondrial membrane. As previously mentioned, under normal conditions, mitochondrial ATP is generated primarily through oxidation of fatty acid and glucose. When transported inside mitochondria as metabolic intermediates, these substrates produce nicotinamide adenine dinucleotide (NADH), reduced flavin adenine dinucleotide (FADH_2_), and GTP through the Krebs cycle. NADH and FADH_2_ actually transport redox energy to the electron transport chain (ETC), which is then used to generate a proton gradient for ATP synthesis [117]. Meanwhile, mitochondrial reactive oxygen species (mt-ROS) are physiologically generated, mainly from complexes I and III of the ETC [118]. At low levels, mt-ROS act as intracellular messengers during cardiac remodeling, whereas at high levels they are responsible for damage to mitochondrial DNA (mt-DNA) and proteins; this in turn impairs transcription of mitochondrial genes coding for components of the ETC, affecting energy production [119].

It has been demonstrated that angiotensin II increases mt-ROS in mice, contributing to cardiac fibrosis and hypertrophy, both of which are crucial for cardiac remodeling, as discussed above. Interestingly, both fibrosis and hypertrophy are reduced in Ang-II-treated genetic mice overexpressing mitochondrial catalases, suggesting that antioxidant therapies may prevent cardiac remodeling [120]. In the same study, the authors showed that mt-ROS-induced cardiac remodeling is mediated by the activation of ERK1/2. Recently, Sirtuin 4 overexpression was found to exacerbate cardiac hypertrophy induced by angiotensin-II, and to impair cardiac function through an increase of mt-ROS and a concomitant reduction of manganese superoxide dismutase (MnSOD) [121]. Moreover, Shiomi et al. demonstrated a reduction in LV remodeling after myocardial infarction in transgenic mice overexpressing glutathione peroxidase, an enzyme that reduces ROS [122]. Similarly, overexpression of peroxiredoxin-3, a mitochondrial antioxidant protein, has also been shown to improve mitochondrial function, reduce cardiac fibrosis and myocyte hypertrophy, and ameliorate LV function [123]. Interestingly, in vivo activation of mitochondrial aldehyde dehydrogenase 2 (ALDH2), a protein involved in detoxifying mitochondrial reactive aldehydes generated during oxidative stress, was shown to be able to rescue pathological ventricular remodeling after MI by reducing myocardial fibrosis and hypertrophy and by restoring mitochondrial function [124].

Excessive ROS production does not represent the only feature of mitochondrial dysfunction. It has been demonstrated that during progression of cardiac remodeling, there is significant downregulation of genes involved in mitochondrial biogenesis, such as PGC-1*α* and PGC-1*β*, p38-MAPK, and mitochondrial transcription factor A (TFAM). For example, mice lacking PGC-1*α* displayed a more rapid progression towards heart failure after transverse aortic constriction [125]. Similarly, PGC-1*β* was shown to be responsible for mitochondrial dysfunction resulting from accelerated myocardial hypertrophy following pressure overload [126]. Furthermore, expression of p38-MAPK was found to be reduced after MI, leading to an impaired capability to oxidize fatty acids, which in turn contributes to LV dilatation [127]. Interestingly, in vivo overexpression of TFAM in a mouse model of MI improved mt-DNA copy number and mitochondrial complex activity, while reducing myocyte hypertrophy, interstitial fibrosis, apoptosis, and chamber dilatation, thus slowing down the overall progression of LV remodeling [128].

## 7. Autophagy Dysregulation and Apoptosis

Autophagy is an evolutionarily conserved mechanism for cellular homeostasis in which the macromolecular constituents of protein and mitochondria are turned over and recycled for energy production, protein synthesis, and the biogenesis of organelles [129]. Autophagy allows the sequestration of portions of cytoplasm by double membrane vesicles called autophagosomes that deliver their content to lysosomes for ultimate digestion [130]. Autophagic regulation relies on both internal and external stimuli, including inflammatory signals such as TNF*α*, which triggers the NF-*κ*B pathway, and DAMPs, which signal through intra- and extracellular PRRs [131]. It has been recently demonstrated that unmethylated mt-DNA that has evaded autophagy is recognized as a DAMP by TLR-9, whose deletion is protective in a TAC model of pressure overload [132].

It has been shown that induction of autophagy exerts cardioprotective effects in several cardiovascular pathologies. In fact, autophagy represents an adaptive mechanism adopted by the heart in response to stress conditions. However, prolonged states of high activation may be detrimental [133]. Autophagy is upregulated during MI as an adaptive response to nutrient deprivation [134], oxidative stress [78], and hypoxia [135]. Cardiac remodeling is reduced in a mouse model of I/R with impaired autophagy through Beclin-1 heterozygous deficiency, compared to wild-type [136]. Interestingly, Zue and colleagues have shown in a model of pressure overload that modulation of autophagy, achieved through Beclin-1 deletion and overexpression, improved or exacerbated pathological remodeling, respectively [137]. Moreover, in a TAC pressure-overload murine model, the administration of pleiotropic HDAC inhibitors, such as trichostatin A, was found to suppress autophagy and attenuate cardiac hypertrophy, suggesting that these are inversely correlated processes [138]. In fact, cardiac-specific deficiency of Atg5 (autophagy-related 5) in mice [139] or *β*-adrenergic stimulation, facilitates myocardial hypertrophy [140], whereas rapamycin-induced autophagic activation can prevent it [141]. Studies on the role of autophagy in cardiovascular diseases have proven that intensity, duration, and contingent activation of autophagy with other signaling pathways are key determinants in cardiac response to pathogenic insults.

Many signaling pathways are involved in the regulation of autophagic flux. For example, we have previously shown that mTOR signaling, a strong negative regulator of autophagy, represents the main molecular switch through which autophagy is inhibited [142]. In fact, mTOR inhibition through rapamycin, everolimus, or lentivirus-mediated overexpression of miR-99a induces autophagy and mitigates cardiac remodeling, whereas autophagic flux inhibition with bafilomycin A1 aggravates post-MI dysfunction and remodeling [143–145]. Interestingly, inhibition of AMPK, a well-known mTORC-1 upstream negative regulator that senses cytoplasmic AMP concentration, was also found to impair autophagy via an increased interaction between B-cell lymphoma (BCL)-2 and Beclin-1 [142]. This is consistent with our previous study demonstrating the same mechanism for Mst-1-dependent autophagy suppression [78].

As discussed above, Mst-1 appears to be a fundamental link between autophagy and apoptosis. In fact, it was previously shown that Mst-1 is inhibited by mTORC-2, thereby improving cardiac response to stress [75]. Recent studies have shown that although excessive activation of autophagy may lead to cell death, normal physiologic activation actually protects cells from apoptotic death [146]. Indeed extensive crosstalk has been reported between autophagy and apoptosis in the adult myocardium. The opposing nature of these two phenomena is based on the interaction between Beclin-1 and Bcl-2 family members, whose phosphorylated active forms inhibit mitochondria outer membrane permeabilization (MOMP), consequently preventing initiation of the apoptotic intrinsic pathway. Beclin-1 is part of a class III PI(3)K complex and, along with VPS-34 and VPS-15, is responsible for the formation of autophagic vesicles. Phosphorylation of Bcl-2 by several regulatory kinases, such as JNK, strongly reduces Bcl-2 affinity for Beclin-1, resulting in the interruption of the sequestration process that inhibits autophagy [147, 148]. Moreover, apoptosis activation impairs autophagy through direct caspase-mediated cleavage of Atg-4D and Atg-5 [129, 133]. Lastly, AMPK suppression results in the activation of mTORC1. This in turn phosphorylates and inhibits ULK-1, an autophagy activator upstream of the class III PI3K complex [149].

Progressive cell death in the chronically overloaded heart is considered among the leading causes of cardiac remodeling [150]. Several cytokines, through an increase in ROS levels and GPCR signaling, can trigger apoptosis in the failing ischemic or overloaded heart [119]. A plethora of GPCRs converges on kinases, such as Ask1, p38-MAPK, JNK, PKC, and CAMKII. CAMKII acts as a crosslink between calcium dysregulation and ROS production [151]. Moreover, CAMKII is additionally activated by ROS and is upregulated downstream of AT-II GPCR signaling by NOX-4 [152]. However, the activity of previously mentioned factors (e.g., AKT, PIM-1, GSK-3*β*) can counteract proapoptotic stimuli [153].

Recently, a novel form of controlled cell death, termed programmed necrosis or “necroptosis,” has shown a prominent role in many pathologies, including cardiovascular diseases. It is described by loss of cytoplasmic and mitochondrial membrane integrity, with a consequent dispersion of DAMPs and other proinflammatory stimuli [154].

## 8. Clinical and Translational Perspectives

To date, several drugs are already known to exert beneficial effects in cardiac remodeling, slowing progression towards heart failure [155]. Nonetheless, novel targets and strategies are needed to expand therapeutic options and to increase biological and clinical efficacies. Since the early nineties, a variety of randomized clinical trials has demonstrated a beneficial effect of ACE inhibitors, mineralocorticoid receptor blockers, and angiotensin receptor blockers (ARBs) [155]. These inhibitors of the RAAS act at different points of the signaling cascade of angiotensin II, which can induce cardiac remodeling independently of changes in blood pressure [156]. Nevertheless, control of blood pressure remains an important protective therapeutic strategy after MI. Unfortunately, other agents have been less successful in the clinical setting. The vasopressin antagonist tolvaptan improved patient symptoms in the EVEREST trial but ultimately did not improve long-term mortality or HF-related morbidity [157]. Similarly, endothelin-1 is thought to have a cardiac hypertrophic effect through transcriptional and posttranslational modifications, increasing cardiomyocyte growth and contractility [158]. Yet endothelin-1 antagonists have not shown mortality benefit in the ENCOR, RITZ-4, and EARTH clinical trials [159–161].

*β*-adrenergic receptor blockers are also extensively used to reduce adverse cardiac remodeling, although controversial results have emerged from clinical trials [162]. Treatment with *β*-blocking agents opposes adverse remodeling at both the molecular and organ levels [163–165]. A recent report has also shown a significant clinical and biological correlation between *β*-blocker treatments in patients and features of reduced profibrotic potential of resident cardiac progenitor cells [166]. Interestingly, excessive adrenergic drive in situ may also affect the myofibroblast potential of resident progenitors through *β*2-signaling [167], contributing to detrimental profibrotic conditions. Resident progenitors are known to contribute to cardiac homeostasis and can be exploited for therapeutic purposes [168–170]. In fact, cardiac cell therapy using resident progenitors has also been shown to exert therapeutic effects through paracrine antifibrotic mechanisms [171]. These studies highlight how *β*-blockers may act at multiple levels and on different mechanisms of fibrosis and remodeling. They also suggest how different approaches, such as *β*-blockers and regenerative therapy, may be integrated to obtain adjuvant or synergic effects.

Other strategies can also be developed to increase the efficacy of *β*-blockers. In fact, experimental gene therapy with an engineered catalytically inactive G-protein receptor kinase-2 (*β*-ARK_ct_) reduced *β*-receptor internalization and degradation, augmenting *β*-blocker effects in a rodent model of heart failure [172, 173]. The same was reported in failing human myocytes [174].

Nonetheless, since cardiac remodeling is a complex multifactorial process, gene therapy directed to single genes may not be efficient enough in clinical settings. Interestingly, different combined approaches based on transcription factors and miRs are showing encouraging results. For example, antago-miR mediated inactivation of miR-25, which is selectively upregulated in cardiomyocytes from TAC-overloaded hearts and targets mRNAs such as sarcoplasmic reticulum calcium ATPase 2a (SERCA2a) and inositol-3′-phosphate receptor-1 (IP3R1), improves calcium reuptake and myocardial contractility during HF [175]. The same effect has been obtained through adenoviral overexpression of SERCA2a [176]. Based on this rationale, and considering the increased mortality reported in long-term treatment with positive inotropic agents, an adeno-associated virus AAV1/SERCA2a was created and used in the CUPID clinical trial, with long-term safety and efficacy [177].

Gene transfer has also been therapeutically explored to achieve neocardiomyogenesis. In fact, overexpression of the oncogenic miR-17 to miR-92 cluster was sufficient to induce cardiomyocyte proliferation [178]. Moreover, direct fibroblast reprogramming into beating cardiomyocyte-like cells was performed through concomitant gene transfer of GATA-4, heart and neural crest derivatives-expressed protein (HAND)-2, T-box transcription factor (TBX)-5, and MEF-2 [179]. In vivo gene transfer of these transcription factors after MI attenuated fibrosis and cardiac dysfunction [179]. Interestingly, it was recently shown that hypertrophy and fibrosis could also be treated with the administration of epigenetic drugs, such as the DNA methylation inhibitor 5-azacytidine or the previously mentioned HDAC inhibitors [180].

In conclusion, despite the promising strategies that have been proposed and developed, a collective and integrated translational effort is needed to find the most effective and safe strategy to reach the ambitious goal of successfully treating cardiac remodeling.

## References

[1] Hochman J. S., Bulkley B. H. (1982). Expansion of acute myocardial infarction: an experimental study. *Circulation*.

[2] Pfeffer J. M., Pfeffer M. A., Braunwald E. (1985). Influence of chronic captopril therapy on the infarcted left ventricle of the rat. *Circulation Research*.

[3] Frangogiannis N. G. (2012). Regulation of the inflammatory response in cardiac repair. *Circulation Research*.

[4] Lehnart S. E., Maier L. S., Hasenfuss G. (2009). Abnormalities of calcium metabolism and myocardial contractility depression in the failing heart. *Heart Failure Reviews*.

[5] Sciarretta S., Paneni F., Palano F. (2009). Role of the renin-angiotensin-aldosterone system and inflammatory processes in the development and progression of diastolic dysfunction. *Clinical Science (London, England : 1979)*.

[6] Goldberg L. R. (2010). In the clinic. Heart failure. *Annals of Internal Medicine*.

[7] Cohn J. N., Ferrari R., Sharpe N. (2000). Cardiac remodeling—concepts and clinical implications: a consensus paper from an international forum on cardiac remodeling. Behalf of an international forum on cardiac remodeling. *Journal of the American College of Cardiology*.

[8] Nian M., Lee P., Khaper N., Liu P. (2004). Inflammatory cytokines and postmyocardial infarction remodeling. *Circulation Research*.

[9] Swynghedauw B. (1999). Molecular mechanisms of myocardial remodeling. *Physiological Reviews*.

[10] Dorn G. W. (2007). The fuzzy logic of physiological cardiac hypertrophy. *Hypertension*.

[11] Opie L. H., Commerford P. J., Gersh B. J., Pfeffer M. A. (2006). Controversies in ventricular remodelling. *Lancet*.

[12] Hill J. A., Olson E. N. (2008). Cardiac plasticity. *The New England Journal of Medicine*.

[13] Burchfield J. S., Xie M., Hill J. A. (2013). Pathological ventricular remodeling: mechanisms: part 1 of 2. *Circulation*.

[14] Ohtani T., Mohammed S. F., Yamamoto K. (2012). Diastolic stiffness as assessed by diastolic wall strain is associated with adverse remodelling and poor outcomes in heart failure with preserved ejection fraction. *European Heart Journal*.

[15] Harvey P. A., Leinwand L. A. (2011). The cell biology of disease: cellular mechanisms of cardiomyopathy. *The Journal of Cell Biology*.

[16] Benard L., Oh J. G., Cacheux M. (2016). Cardiac Stim1 silencing impairs adaptive hypertrophy and promotes heart failure through inactivation of mTORC2/Akt signaling. *Circulation*.

[17] Carnevale D., Cifelli G., Mascio G. (2011). Placental growth factor regulates cardiac inflammation through the tissue inhibitor of metalloproteinases-3/tumor necrosis factor-alpha-converting enzyme axis: crucial role for adaptive cardiac remodeling during cardiac pressure overload. *Circulation*.

[18] Pacini L., Suffredini S., Ponti D. (2013). Altered calcium regulation in isolated cardiomyocytes from Egr-1 knock-out mice. *Canadian Journal of Physiology and Pharmacology*.

[19] Shah A. M., Mann D. L. (2011). In search of new therapeutic targets and strategies for heart failure: recent advances in basic science. *Lancet*.

[20] Matsui T., Li L., Wu J. C. (2002). Phenotypic spectrum caused by transgenic overexpression of activated Akt in the heart. *The Journal of Biological Chemistry*.

[21] Shiojima I., Sato K., Izumiya Y. (2005). Disruption of coordinated cardiac hypertrophy and angiogenesis contributes to the transition to heart failure. *Journal of Clinical Investigation*.

[22] Luckey S. W., Walker L. A., Smyth T. (2009). The role of Akt/GSK-3beta signaling in familial hypertrophic cardiomyopathy. *Journal of Molecular and Cellular Cardiology*.

[23] Michael A., Haq S., Chen X. (2004). Glycogen synthase kinase-3beta regulates growth, calcium homeostasis, and diastolic function in the heart. *The Journal of Biological Chemistry*.

[24] Sadoshima J., Izumo S. (1993). Mechanical stretch rapidly activates multiple signal transduction pathways in cardiac myocytes: potential involvement of an autocrine/paracrine mechanism. *The EMBO Journal*.

[25] Molkentin J. D., Lu J. R., Antos C. L. (1998). A calcineurin-dependent transcriptional pathway for cardiac hypertrophy. *Cell*.

[26] Backs J., Olson E. N. (2006). Control of cardiac growth by histone acetylation/deacetylation. *Circulation Research*.

[27] Dassanayaka S., Jones S. P. (2015). Recent developments in heart failure. *Circulation Research*.

[28] Appari M., Breitbart A., Brandes F. (2017). C1q-TNF-related protein-9 promotes cardiac hypertrophy and failure. *Circulation Research*.

[29] Herron T. J., McDonald K. S. (2002). Small amounts of alpha-myosin heavy chain isoform expression significantly increase power output of rat cardiac myocyte fragments. *Circulation Research*.

[30] Pokreisz P., Vandenwijngaert S., Bito V. (2009). Ventricular phosphodiesterase-5 expression is increased in patients with advanced heart failure and contributes to adverse ventricular remodeling after myocardial infarction in mice. *Circulation*.

[31] Takimoto E., Champion H. C., Li M. (2005). Chronic inhibition of cyclic GMP phosphodiesterase 5A prevents and reverses cardiac hypertrophy. *Nature Medicine*.

[32] Matsushima S., Kuroda J., Ago T. (2013). Increased oxidative stress in the nucleus caused by Nox4 mediates oxidation of HDAC4 and cardiac hypertrophy. *Circulation Research*.

[33] Toko H., Konstandin M. H., Doroudgar S. (2013). Regulation of cardiac hypertrophic signaling by prolyl isomerase Pin1. *Circulation Research*.

[34] Sano M., Minamino T., Toko H. (2007). p53-induced inhibition of Hif-1 causes cardiac dysfunction during pressure overload. *Nature*.

[35] Zhang M., Brewer A. C., Schröder K. (2010). NADPH oxidase-4 mediates protection against chronic load-induced stress in mouse hearts by enhancing angiogenesis. *Proceedings of the National Academy of Sciences of the United States of America*.

[36] Segura A. M., Frazier O. H., Buja L. M. (2014). Fibrosis and heart failure. *Heart Failure Reviews*.

[37] Prabhu S. D., Frangogiannis N. G. (2016). The biological basis for cardiac repair after myocardial infarction. *Circulation Research*.

[38] Kandalam V., Basu R., Moore L. (2011). Lack of tissue inhibitor of metalloproteinases 2 leads to exacerbated left ventricular dysfunction and adverse extracellular matrix remodeling in response to biomechanical stress. *Circulation*.

[39] Kong P., Christia P., Frangogiannis N. G. (2014). The pathogenesis of cardiac fibrosis. *Cellular and Molecular Life Sciences*.

[40] Wang J., Chen H., Seth A., Mcculloch C. A. (2003). Mechanical force regulation of myofibroblast differentiation in cardiac fibroblasts. *American Journal of Physiology. Heart and Circulatory Physiology*.

[41] Zeisberg E. M., Tarnavski O., Zeisberg M. (2007). Endothelial-to-mesenchymal transition contributes to cardiac fibrosis. *Nature Medicine*.

[42] Travers J. G., Kamal F. A., Robbins J., Yutzey K. E., Blaxall B. C. (2016). Cardiac fibrosis: the fibroblast awakens. *Circulation Research*.

[43] Chen C. W., Okada M., Proto J. D. (2013). Human pericytes for ischemic heart repair. *Stem Cells*.

[44] Katare R., Riu F., Mitchell K. (2011). Transplantation of human pericyte progenitor cells improves the repair of infarcted heart through activation of an angiogenic program involving micro-RNA-132. *Circulation Research*.

[45] Mollmann H., Nef H. M., Kostin S. (2006). Bone marrow-derived cells contribute to infarct remodelling. *Cardiovascular Research*.

[46] Ruiz-Villalba A., Simón A. M., Pogontke C. (2015). Interacting resident epicardium-derived fibroblasts and recruited bone marrow cells form myocardial infarction scar. *Journal of the American College of Cardiology*.

[47] Yano T., Miura T., Ikeda Y. (2005). Intracardiac fibroblasts, but not bone marrow derived cells, are the origin of myofibroblasts in myocardial infarct repair. *Cardiovascular Pathology*.

[48] Fujita S., Shimojo N., Terasaki F. (2013). Atrial natriuretic peptide exerts protective action against angiotensin II-induced cardiac remodeling by attenuating inflammation via endothelin-1/endothelin receptor A cascade. *Heart and Vessels*.

[49] Mortensen R. M. (2012). Immune cell modulation of cardiac remodeling. *Circulation*.

[50] Saxena A., Saxena A., Dobaczewski M. (2014). Regulatory T cells are recruited in the infarcted mouse myocardium and may modulate fibroblast phenotype and function. *American Journal of Physiology. Heart and Circulatory Physiology*.

[51] Levick S. P., McLarty J. L., Murray D. B., Freeman R. M., Carver W. E., Brower G. L. (2009). Cardiac mast cells mediate left ventricular fibrosis in the hypertensive rat heart. *Hypertension*.

[52] Zhang W., Chancey A. L., Tzeng H. P. (2011). The development of myocardial fibrosis in transgenic mice with targeted overexpression of tumor necrosis factor requires mast cell-fibroblast interactions. *Circulation*.

[53] Barouch L. A., Gao D., Chen L. (2006). Cardiac myocyte apoptosis is associated with increased DNA damage and decreased survival in murine models of obesity. *Circulation Research*.

[54] Privratsky J. R., Wold L. E., Sowers J. R., Quinn M. T., Ren J. (2003). AT1 blockade prevents glucose-induced cardiac dysfunction in ventricular myocytes: role of the AT1 receptor and NADPH oxidase. *Hypertension*.

[55] Chen K., Mehta J. L., Li D., Joseph L., Joseph J. (2004). Transforming growth factor beta receptor endoglin is expressed in cardiac fibroblasts and modulates profibrogenic actions of angiotensin II. *Circulation Research*.

[56] Wang W., Huang X. R., Canlas E. (2006). Essential role of Smad3 in angiotensin II-induced vascular fibrosis. *Circulation Research*.

[57] Biernacka A., Dobaczewski M., Frangogiannis N. G. (2011). TGF-*β* signaling in fibrosis. *Growth Factors*.

[58] Li X., Zhao D., Guo Z. (2016). Overexpression of SerpinE2/protease nexin-1 contribute to pathological cardiac fibrosis via increasing collagen deposition. *Scientific Reports*.

[59] Zhang J., Fan G., Zhao H. (2017). Targeted inhibition of focal adhesion kinase attenuates cardiac fibrosis and preserves heart function in adverse cardiac remodeling. *Scientific Reports*.

[60] Mehta P. K., Griendling K. K. (2007). Angiotensin II cell signaling: physiological and pathological effects in the cardiovascular system. *American Journal of Physiology. Cell Physiology*.

[61] Deschamps A. M., Spinale F. G. (2006). Pathways of matrix metalloproteinase induction in heart failure: bioactive molecules and transcriptional regulation. *Cardiovascular Research*.

[62] Blaser H., Dostert C., Mak T. W., Brenner D. (2016). TNF and ROS crosstalk in inflammation. *Trends in Cell Biology*.

[63] Xie J., Méndez J. D., Méndez-Valenzuela V., Aguilar-Hernández M. M. (2013). Cellular signalling of the receptor for advanced glycation end products (RAGE). *Cellular Signalling*.

[64] Rojas A., Delgado-López F., González I., Pérez-Castro R., Romero J., Rojas I. (2013). The receptor for advanced glycation end-products: a complex signaling scenario for a promiscuous receptor. *Cellular Signalling*.

[65] Volz H. C., Laohachewin D., Seidel C. (2012). S100A8/A9 aggravates post-ischemic heart failure through activation of RAGE-dependent NF-kappaB signaling. *Basic Research in Cardiology*.

[66] Oldfield M. D., Bach L. A., Forbes J. M. (2001). Advanced glycation end products cause epithelial-myofibroblast transdifferentiation via the receptor for advanced glycation end products (RAGE). *Journal of Clinical Investigation*.

[67] Umadevi S., Gopi V., Elangovan V. (2014). Regulatory mechanism of gallic acid against advanced glycation end products induced cardiac remodeling in experimental rats. *Chemico-Biological Interactions*.

[68] Zhao L., Zhang W., Wang L. P., Li G. R., Deng X. L. (2012). Advanced glycation end products promote proliferation of cardiac fibroblasts by upregulation of KCa3.1 channels. *Pflügers Archiv: European Journal of Physiology*.

[69] Pesce M., Messina E., Chimenti I., Beltrami A. P. (2017). Cardiac mechanoperception: a life-long story from early beats to aging and failure. *Stem Cells and Development*.

[70] Zhou Q., Li L., Zhao B., Guan K. L. (2015). The hippo pathway in heart development, regeneration, and diseases. *Circulation Research*.

[71] Dhingra R., Kirshenbaum L. A. (2013). Mst-1 switches between cardiac cell life and death. *Nature Medicine*.

[72] Wang W., Xiao Z. D., Li X. (2015). AMPK modulates hippo pathway activity to regulate energy homeostasis. *Nature Cell Biology*.

[73] Yu F. X., Zhao B., Panupinthu N. (2012). Regulation of the hippo-YAP pathway by G-protein-coupled receptor signaling. *Cell*.

[74] Wennmann D. O., Vollenbröker B., Eckart A. K. (2014). The hippo pathway is controlled by angiotensin II signaling and its reactivation induces apoptosis in podocytes. *Cell Death & Disease*.

[75] Sciarretta S., Zhai P., Maejima Y. (2015). mTORC2 regulates cardiac response to stress by inhibiting MST1. *Cell Reports*.

[76] Del Re D. P., Matsuda T., Zhai P. (2014). Mst1 promotes cardiac myocyte apoptosis through phosphorylation and inhibition of Bcl-xL. *Molecular Cell*.

[77] Ikeda Y., Sciarretta S., Nagarajan N. (2014). New insights into the role of mitochondrial dynamics and autophagy during oxidative stress and aging in the heart. *Oxidative Medicine and Cellular Longevity*.

[78] Maejima Y., Kyoi S., Zhai P. (2013). Mst1 inhibits autophagy by promoting the interaction between Beclin1 and Bcl-2. *Nature Medicine*.

[79] Saito T., Sadoshima J. (2015). Molecular mechanisms of mitochondrial autophagy/mitophagy in the heart. *Circulation Research*.

[80] Yamamoto S., Yang G., Zablocki D. (2003). Activation of Mst1 causes dilated cardiomyopathy by stimulating apoptosis without compensatory ventricular myocyte hypertrophy. *The Journal of Clinical Investigation*.

[81] Roncarati R., Viviani Anselmi C., Losi M. A. (2014). Circulating miR-29a, among other up-regulated microRNAs, is the only biomarker for both hypertrophy and fibrosis in patients with hypertrophic cardiomyopathy. *Journal of the American College of Cardiology*.

[82] van Rooij E., Sutherland L. B., Thatcher J. E. (2008). Dysregulation of microRNAs after myocardial infarction reveals a role of miR-29 in cardiac fibrosis. *Proceedings of the National Academy of Sciences of the United States of America*.

[83] Castoldi G., Di Gioia C. R., Bombardi C. (2012). MiR-133a regulates collagen 1A1: potential role of miR-133a in myocardial fibrosis in angiotensin II-dependent hypertension. *Journal of Cellular Physiology*.

[84] Cavarretta E., Condorelli G. (2015). miR-21 and cardiac fibrosis: another brick in the wall?. *European Heart Journal*.

[85] Cavarretta E., Frati G. (2016). MicroRNAs in coronary heart disease: ready to enter the clinical arena?. *BioMed Research International*.

[86] Neubauer S. (2007). The failing heart—an engine out of fuel. *The New England Journal of Medicine*.

[87] Kantor P. F., Lucien A., Kozak R., Lopaschuk G. D. (2000). The antianginal drug trimetazidine shifts cardiac energy metabolism from fatty acid oxidation to glucose oxidation by inhibiting mitochondrial long-chain 3-ketoacyl coenzyme A thiolase. *Circulation Research*.

[88] Frati G., Schirone L., Chimenti I. (2017). An overview of the inflammatory signalling mechanisms in the myocardium underlying the development of diabetic cardiomyopathy. *Cardiovascular Research*.

[89] Desvergne B., Wahli W. (1999). Peroxisome proliferator-activated receptors: nuclear control of metabolism. *Endocrine Reviews*.

[90] Puigserver P., Spiegelman B. M. (2003). Peroxisome proliferator-activated receptor-gamma coactivator 1 alpha (PGC-1 alpha): transcriptional coactivator and metabolic regulator. *Endocrine Reviews*.

[91] Duncan J. G., Fong J. L., Medeiros D. M., Finck B. N., Kelly D. P. (2007). Insulin-resistant heart exhibits a mitochondrial biogenic response driven by the peroxisome proliferator-activated receptor-alpha/PGC-1alpha gene regulatory pathway. *Circulation*.

[92] Finck B. N., Kelly D. P. (2002). Peroxisome proliferator-activated receptor alpha (PPARalpha) signaling in the gene regulatory control of energy metabolism in the normal and diseased heart. *Journal of Molecular and Cellular Cardiology*.

[93] Sano M., Wang S. C., Shirai M. (2004). Activation of cardiac Cdk9 represses PGC-1 and confers a predisposition to heart failure. *The EMBO Journal*.

[94] Duncan J. G., Finck B. N. (2008). The PPARalpha-PGC-1alpha Axis controls cardiac energy metabolism in healthy and diseased myocardium. *PPAR Research*.

[95] Arany Z., He H., Lin J. (2005). Transcriptional coactivator PGC-1 alpha controls the energy state and contractile function of cardiac muscle. *Cell Metabolism*.

[96] Chen Y., Wang Y., Chen J. (2012). Roles of transcriptional corepressor RIP140 and coactivator PGC-1alpha in energy state of chronically infarcted rat hearts and mitochondrial function of cardiomyocytes. *Molecular and Cellular Endocrinology*.

[97] Hsieh M. C., Das D., Sambandam N., Zhang M. Q., Nahlé Z. (2008). Regulation of the PDK4 isozyme by the Rb-E2F1 complex. *The Journal of Biological Chemistry*.

[98] Ventura-Clapier R., Garnier A., Veksler V. (2008). Transcriptional control of mitochondrial biogenesis: the central role of PGC-1alpha. *Cardiovascular Research*.

[99] Liu J., Wang P., Luo J. (2011). Peroxisome proliferator-activated receptor beta/delta activation in adult hearts facilitates mitochondrial function and cardiac performance under pressure-overload condition. *Hypertension*.

[100] Liu J., Wang P., He L. (2011). Cardiomyocyte-restricted deletion of PPARbeta/delta in PPARalpha-null mice causes impaired mitochondrial biogenesis and defense, but no further depression of myocardial fatty acid oxidation. *PPAR Research*.

[101] Lehman J. J., Barger P. M., Kovacs A., Saffitz J. E., Medeiros D. M., Kelly D. P. (2000). Peroxisome proliferator-activated receptor gamma coactivator-1 promotes cardiac mitochondrial biogenesis. *The Journal of Clinical Investigation*.

[102] Lehman J. J., Boudina S., Banke N. H. (2008). The transcriptional coactivator PGC-1alpha is essential for maximal and efficient cardiac mitochondrial fatty acid oxidation and lipid homeostasis. *American Journal of Physiology. Heart and Circulatory Physiology*.

[103] Daynes R. A., Jones D. C. (2002). Emerging roles of PPARs in inflammation and immunity. *Nature Reviews. Immunology*.

[104] Buroker N. E., Barboza J., Huang J.-Y. (2009). The IkappaBalpha gene is a peroxisome proliferator-activated receptor cardiac target gene. *The FEBS Journal*.

[105] el Azzouzi H., Leptidis S., Bourajjaj M., van Bilsen M., da Costa Martins P. A., De Windt L. J. (2012). MEK1 inhibits cardiac PPARalpha activity by direct interaction and prevents its nuclear localization. *PloS One*.

[106] Alvarez-Guardia D., Palomer X., Coll T. (2011). PPARbeta/delta activation blocks lipid-induced inflammatory pathways in mouse heart and human cardiac cells. *Biochimica et Biophysica Acta*.

[107] Asakawa M., Takano H., Nagai T. (2002). Peroxisome proliferator-activated receptor gamma plays a critical role in inhibition of cardiac hypertrophy in vitro and in vivo. *Circulation*.

[108] Diep Q. N., Amiri F., Benkirane K., Paradis P., Schiffrin E. L. (2004). Long-term effects of the PPAR&gamma; activator pioglitazone on cardiac inflammation in stroke-prone spontaneously hypertensive rats. *Journal of Controlled Release : Official Journal of the Controlled Release Society*.

[109] Palomer X., Alvarez-Guardia D., Rodríguez-Calvo R. (2009). TNF-alpha reduces PGC-1alpha expression through NF-kappaB and p38 MAPK leading to increased glucose oxidation in a human cardiac cell model. *Cardiovascular Research*.

[110] Alvarez-Guardia D., Palomer X., Coll T. (2010). The p65 subunit of NF-kappaB binds to PGC-1alpha, linking inflammation and metabolic disturbances in cardiac cells. *Cardiovascular Research*.

[111] Meng F., Liu L., Chin P. C., D'Mello S. R. (2002). Akt is a downstream target of NF-kappa B. *Journal of Biological Chemistry*.

[112] Li Y. Y., Chen D., Watkins S. C., Feldman A. M. (2001). Mitochondrial abnormalities in tumor necrosis factor-alpha-induced heart failure are associated with impaired DNA repair activity. *Circulation*.

[113] Oudit G. Y., Sun H., Kerfant B. G., Crackower M. A., Penninger J. M., Backx P. H. (2004). The role of phosphoinositide-3 kinase and PTEN in cardiovascular physiology and disease. *Journal of Molecular and Cellular Cardiology*.

[114] Song X., Kusakari Y., Xiao C. Y. (2010). mTOR attenuates the inflammatory response in cardiomyocytes and prevents cardiac dysfunction in pathological hypertrophy. *American Journal of Physiology. Cell Physiology*.

[115] Lin M. T., Beal M. F. (2006). Mitochondrial dysfunction and oxidative stress in neurodegenerative diseases. *Nature*.

[116] Yu E., Mercer J., Bennett M. (2012). Mitochondria in vascular disease. *Cardiovascular Research*.

[117] Turrens J. F. (2003). Mitochondrial formation of reactive oxygen species. *The Journal of Physiology*.

[118] Balaban R. S., Nemoto S., Finkel T. (2005). Mitochondria, oxidants, and aging. *Cell*.

[119] Burgoyne J. R., Mongue-Din H., Eaton P., Shah A. M. (2012). Redox signaling in cardiac physiology and pathology. *Circulation Research*.

[120] Dai D. F., Johnson S. C., Villarin J. J. (2011). Mitochondrial oxidative stress mediates angiotensin II-induced cardiac hypertrophy and Galphaq overexpression-induced heart failure. *Circulation Research*.

[121] Luo Y. X., Tang X., An X. Z. (2017). SIRT4 accelerates Ang II-induced pathological cardiac hypertrophy by inhibiting manganese superoxide dismutase activity. *European Heart Journal*.

[122] Shiomi T., Tsutsui H., Matsusaka H. (2004). Overexpression of glutathione peroxidase prevents left ventricular remodeling and failure after myocardial infarction in mice. *Circulation*.

[123] Matsushima S., Ide T., Yamato M. (2006). Overexpression of mitochondrial peroxiredoxin-3 prevents left ventricular remodeling and failure after myocardial infarction in mice. *Circulation*.

[124] Gomes K. M., Campos J. C., Bechara L. R. (2014). Aldehyde dehydrogenase 2 activation in heart failure restores mitochondrial function and improves ventricular function and remodelling. *Cardiovascular Research*.

[125] Arany Z., Novikov M., Chin S., Ma Y., Rosenzweig A., Spiegelman B. M. (2006). Transverse aortic constriction leads to accelerated heart failure in mice lacking PPAR-gamma coactivator 1alpha. *Proceedings of the National Academy of Sciences of the United States of America*.

[126] Riehle C., Wende A. R., Zaha V. G. (2011). PGC-1beta deficiency accelerates the transition to heart failure in pressure overload hypertrophy. *Circulation Research*.

[127] Amorim P. A., Nguyen T. D., Shingu Y. (2010). Myocardial infarction in rats causes partial impairment in insulin response associated with reduced fatty acid oxidation and mitochondrial gene expression. *The Journal of Thoracic and Cardiovascular Surgery*.

[128] Ikeuchi M., Matsusaka H., Kang D. (2005). Overexpression of mitochondrial transcription factor a ameliorates mitochondrial deficiencies and cardiac failure after myocardial infarction. *Circulation*.

[129] Levine B., Kroemer G. (2008). Autophagy in the pathogenesis of disease. *Cell*.

[130] Singh R., Cuervo A. M. (2011). Autophagy in the cellular energetic balance. *Cell Metabolism*.

[131] Levine B., Mizushima N., Virgin H. W. (2011). Autophagy in immunity and inflammation. *Nature*.

[132] Oka T., Hikoso S., Yamaguchi O. (2012). Mitochondrial DNA that escapes from autophagy causes inflammation and heart failure. *Nature*.

[133] Sciarretta S., Yee D., Shenoy V., Nagarajan N., Sadoshima J. (2014). The importance of autophagy in cardioprotection. *High Blood Pressure and Cardiovascular Prevention*.

[134] Matsui Y., Takagi H., Qu X. (2007). Distinct roles of autophagy in the heart during ischemia and reperfusion: roles of AMP-activated protein kinase and Beclin 1 in mediating autophagy. *Circulation Research*.

[135] Zhang H., Bosch-Marce M., Shimoda L. A. (2008). Mitochondrial autophagy is an HIF-1-dependent adaptive metabolic response to hypoxia. *The Journal of Biological Chemistry*.

[136] Ma X., Liu H., Foyil S. R. (2012). Impaired autophagosome clearance contributes to cardiomyocyte death in ischemia/reperfusion injury. *Circulation*.

[137] Zhu H., Tannous P., Johnstone J. L. (2007). Cardiac autophagy is a maladaptive response to hemodynamic stress. *The Journal of Clinical Investigation*.

[138] Cao D. J., Wang Z. V., Battiprolu P. K. (2011). Histone deacetylase (HDAC) inhibitors attenuate cardiac hypertrophy by suppressing autophagy. *Proceedings of the National Academy of Sciences of the United States of America*.

[139] Nakai A., Yamaguchi O., Takeda T. (2007). The role of autophagy in cardiomyocytes in the basal state and in response to hemodynamic stress. *Nature Medicine*.

[140] Pfeifer U., Föhr J., Wilhelm W., Dämmrich J. (1987). Short-term inhibition of cardiac cellular autophagy by isoproterenol. *Journal of Molecular and Cellular Cardiology*.

[141] Kuzman J. A., O'Connell T. D., Gerdes A. M. (2007). Rapamycin prevents thyroid hormone-induced cardiac hypertrophy. *Endocrinology*.

[142] Sciarretta S., Volpe M., Sadoshima J. (2014). Mammalian target of rapamycin signaling in cardiac physiology and disease. *Circulation Research*.

[143] Buss S. J., Muenz S., Riffel J. H. (2009). Beneficial effects of mammalian target of rapamycin inhibition on left ventricular remodeling after myocardial infarction. *Journal of the American College of Cardiology*.

[144] Kanamori H., Takemura G., Goto K. (2011). The role of autophagy emerging in postinfarction cardiac remodelling. *Cardiovascular Research*.

[145] Li Q., Xie J., Li R. (2014). Overexpression of microRNA-99a attenuates heart remodelling and improves cardiac performance after myocardial infarction. *Journal of Cellular and Molecular Medicine*.

[146] Biala A. K., Kirshenbaum L. A. (2014). The interplay between cell death signaling pathways in the heart. *Trends in Cardiovascular Medicine*.

[147] Wei Y., Pattingre S., Sinha S., Bassik M., Levine B. (2008). JNK1-mediated phosphorylation of Bcl-2 regulates starvation-induced autophagy. *Molecular Cell*.

[148] Wei Y., Sinha S., Levine B. (2008). Dual role of JNK1-mediated phosphorylation of Bcl-2 in autophagy and apoptosis regulation. *Autophagy*.

[149] Egan D. F., Shackelford D. B., Mihaylova M. M. (2011). Phosphorylation of ULK1 (hATG1) by AMP-activated protein kinase connects energy sensing to mitophagy. *Science*.

[150] Whelan R. S., Kaplinskiy V., Kitsis R. N. (2010). Cell death in the pathogenesis of heart disease: mechanisms and significance. *Annual Review of Physiology*.

[151] Dorn G. W. (2009). Apoptotic and non-apoptotic programmed cardiomyocyte death in ventricular remodelling. *Cardiovascular Research*.

[152] Erickson J. R., Joiner M. L., Guan X. (2008). A dynamic pathway for calcium-independent activation of CaMKII by methionine oxidation. *Cell*.

[153] Muraski J. A., Rota M., Misao Y. (2007). Pim-1 regulates cardiomyocyte survival downstream of Akt. *Nature Medicine*.

[154] Kung G., Konstantinidis K., Kitsis R. N. (2011). Programmed necrosis, not apoptosis, in the heart. *Circulation Research*.

[155] Xie M., Burchfield J. S., Hill J. A. (2013). Pathological ventricular remodeling: therapies: part 2 of 2. *Circulation*.

[156] Ainscough J. F., Drinkhill M. J., Sedo A. (2009). Angiotensin II type-1 receptor activation in the adult heart causes blood pressure-independent hypertrophy and cardiac dysfunction. *Cardiovascular Research*.

[157] Konstam M. A., Gheorghiade M., Burnett J. C. (2007). Effects of oral tolvaptan in patients hospitalized for worsening heart failure: the EVEREST outcome trial. *Jama*.

[158] Sugden P. H. (2003). An overview of endothelin signaling in the cardiac myocyte. *Journal of Molecular and Cellular Cardiology*.

[159] Anand I., McMurray J., Cohn J. N. (2004). Long-term effects of darusentan on left-ventricular remodelling and clinical outcomes in the endothelinA receptor antagonist trial in heart failure (EARTH): randomised, double-blind, placebo-controlled trial. *Lancet*.

[160] Louis A., Cleland J. G., Crabbe S. (2001). Clinical trials update: CAPRICORN, COPERNICUS, MIRACLE, STAF, RITZ-2, RECOVER and RENAISSANCE and cachexia and cholesterol in heart failure. Highlights of the Scientific Sessions of the American College of Cardiology, 2001. *European Journal of Heart Failure*.

[161] O'Connor C. M., Gattis W. A., Adams K. F. (2003). Tezosentan in patients with acute heart failure and acute coronary syndromes: results of the randomized intravenous TeZosentan study (RITZ-4). *Journal of the American College of Cardiology*.

[162] Talameh J. A., McLeod H. L., Adams K. F., Patterson J. H. (2012). Genetic tailoring of pharmacotherapy in heart failure: optimize the old, while we wait for something new. *Journal of Cardiac Failure*.

[163] Koitabashi N., Danner T., Zaiman A. L. (2011). Pivotal role of cardiomyocyte TGF-beta signaling in the murine pathological response to sustained pressure overload. *The Journal of Clinical Investigation*.

[164] Lowes B. D., Gilbert E. M., Abraham W. T. (2002). Myocardial gene expression in dilated cardiomyopathy treated with beta-blocking agents. *The New England Journal of Medicine*.

[165] Reiken S., Wehrens X. H., Vest J. A. (2003). Beta-blockers restore calcium release channel function and improve cardiac muscle performance in human heart failure. *Circulation*.

[166] Chimenti I., Pagano F., Cavarretta E. (2016). Beta-blockers treatment of cardiac surgery patients enhances isolation and improves phenotype of cardiosphere-derived cells. *Scientific Reports*.

[167] Pagano F., Angelini F., Siciliano C. (2017). Beta2-adrenergic signaling affects the phenotype of human cardiac progenitor cells through EMT modulation. *Pharmacological Research*.

[168] Chimenti I., Forte E., Angelini F., Giacomello A., Messina E. (2012). From ontogenesis to regeneration: learning how to instruct adult cardiac progenitor cells. *Progress in Molecular Biology and Translational Science*.

[169] Gaetani R., Barile L., Forte E. (2009). New perspectives to repair a broken heart. *Cardiovascular & Hematological Agents in Medicinal Chemistry*.

[170] Peruzzi M., De Falco E., Abbate A. (2015). State of the art on the evidence base in cardiac regenerative therapy: overview of 41 systematic reviews. *BioMed Research International*.

[171] Chimenti I., Smith R. R., Li T. S. (2010). Relative roles of direct regeneration versus paracrine effects of human cardiosphere-derived cells transplanted into infarcted mice. *Circulation Research*.

[172] Petrofski J. A., Koch W. J. (2003). The beta-adrenergic receptor kinase in heart failure. *Journal of Molecular and Cellular Cardiology*.

[173] Rockman H. A., Koch W. J., Lefkowitz R. J. (2002). Seven-transmembrane-spanning receptors and heart function. *Nature*.

[174] Williams M. L., Hata J. A., Schroder J. (2004). Targeted beta-adrenergic receptor kinase (betaARK1) inhibition by gene transfer in failing human hearts. *Circulation*.

[175] Wahlquist C., Jeong D., Rojas-Muñoz A. (2014). Inhibition of miR-25 improves cardiac contractility in the failing heart. *Nature*.

[176] Kumarswamy R., Lyon A. R., Volkmann I. (2012). SERCA2a gene therapy restores microRNA-1 expression in heart failure via an Akt/FoxO3A-dependent pathway. *European Heart Journal*.

[177] Zsebo K., Yaroshinsky A., Rudy J. J. (2014). Long-term effects of AAV1/SERCA2a gene transfer in patients with severe heart failure: analysis of recurrent cardiovascular events and mortality. *Circulation Research*.

[178] Chen J., Huang Z. P., Seok H. Y. (2013). Mir-17-92 cluster is required for and sufficient to induce cardiomyocyte proliferation in postnatal and adult hearts. *Circulation Research*.

[179] Song K., Nam Y. J., Luo X. (2012). Heart repair by reprogramming non-myocytes with cardiac transcription factors. *Nature*.

[180] Watson C. J., Horgan S., Neary R. (2016). Epigenetic therapy for the treatment of hypertension-induced cardiac hypertrophy and fibrosis. *Journal of Cardiovascular Pharmacology and Therapeutics*.

